# Advances in Label-Free Detection of Non-Muscle Invasive Bladder Cancer: A Critical Review

**DOI:** 10.3390/bioengineering12080866

**Published:** 2025-08-12

**Authors:** Gabriela Vera, Javier Cerda-Infante, Mario I. Fernández, Miguel Sánchez-Encinas, Pablo A. Rojas

**Affiliations:** 1 Servicio de Urología, Complejo Asistencial Dr. Sotero del Rio, Santiago 8150215, Chile; gp.veravarela@gmail.com; 2 Environ SPA, Santiago 7750000, Chile; javier@environ.bio; 3 Departamento de Urología, Clínica Alemana Universidad del Desarrollo, Santiago 7610315, Chile; mfernandeza@alemana.cl; 4 Servicio de Urología, Hospital Universitario Rey Juan Carlos, 28933 Madrid, Spain; miguel.sencinas@quironsalud.es; 5 Facultad Ciencias de la Salud, Universidad Rey Juan Carlos, 28933 Madrid, Spain; 6 Instituto de Investigación Sanitaria Fundación Jiménez Díaz (IIS-FJD, UAM), 28040 Madrid, Spain

**Keywords:** non-muscle invasive bladder cancer, label-free biosensors, Raman spectroscopy, SERS, urinary biomarkers, electrochemical biosensor, interferometric flow cytometry

## Abstract

Non-muscle invasive bladder cancer (NMIBC) accounts for about 75% of new bladder cancer diagnoses. Early detection improves survival, yet routine white-light cystoscopy is invasive, costly, and can miss up to 45% of flat or small lesions. These shortcomings have prompted development of label-free diagnostic tools that read the intrinsic optical, electrical, or mechanical signatures of urinary biomarkers without added labels. This review examines recent engineering advances in such platforms for NMIBC detection, focusing on analytical performance, readiness for clinical translation, and remaining barriers to adoption. We compare each technology with conventional cytology using key metrics such as limit of detection, diagnostic accuracy, analysis time, cohort size, and stage of clinical development. Surface-enhanced Raman spectroscopy and interferometric flow cytometry offer femtomolar sensitivity and more than 98% accuracy within minutes, while compact electrochemical sensors targeting NMP22, Galectin-1, and microRNAs reach sub-picogram levels on disposable chips. Standardized sample handling, multicenter validation, and robust cost-effectiveness data are now essential for these tools to advance point-of-care NMIBC surveillance.

## 1. Introduction

Bladder cancer (BC) is the most common neoplasm of the urinary tract and the ninth most frequent cancer worldwide [1]. Limitations in a timely diagnosis of BC are primarily associated with the lack of screening tools and, in some centers, limited access to cystoscopy, which remains the standard of care for BC diagnosis [2].

White-light cystoscopy (WLC), although standard for NMIBC diagnosis, has reduced sensitivity for carcinoma in situ (CIS), with reported values between 58% and 68% [3]. This limitation leads to a significant number of missed lesions and is especially problematic given the aggressive potential of CIS. WLC also lacks specificity, as it cannot reliably distinguish malignant tissue from benign or inflammatory changes. These shortcomings underscore the clinical need for more sensitive, non-invasive strategies such as label-free optical or biochemical detection platforms.

At the time of diagnosis, approximately 75% of cases correspond to non-muscle invasive bladder cancer (NMIBC) [4]. Timely diagnosis is critical, as patients with NMIBC have higher long-term survival rates and a lower risk of cancer-specific mortality compared to those with muscle-invasive disease [5].

Therefore, several alternatives to cystoscopy have been developed for the BC detection. One such method is urinary cytology, which can also be used to monitor patients diagnosed with BC. Despite the advantage of being non-invasive and requiring only a urine sample, urinary cytology has a sensitivity of only 54% [6]; however, this varies by tumor grade—79% in high-grade tumors and only 16% in low-grade tumors [7].

These challenges have underscored the need for novel diagnostic tools that are non-invasive, sensitive, swift, cost-effective, and suitable for a variety of clinical settings. In this context, label-free detection technologies have attracted increasing interest. Unlike traditional methods that rely on fluorescent, enzymatic, or radioactive markers, label-free approaches directly detect biomolecules or other targets based on their intrinsic physical or chemical properties, such as impedance, refractive index, mass, or natural fluorescence. This reduces costs, simplifies workflows, and enhances reproducibility—features that are particularly advantageous for implementation in portable or point-of-care devices [8].

Label-free detection strategies have emerged as promising alternatives to conventional diagnostic methods by enabling the identification of cancer-associated features without the need for fluorescent or radioactive labels. These approaches fall into two distinct but complementary categories: biosensors, which detect molecular biomarkers such as nucleic acids, proteins, or extracellular vesicles in urine, and cellular or optical methods, which analyze intact exfoliated cells or vesicles using spectroscopic or interferometric principles. While both strategies aim to enable earlier and more accessible diagnosis of NMIBC, they differ substantially in detection principles, technical requirements, and potential for clinical integration.

These technologies have demonstrated the ability to identify exfoliated tumor cells, circulating DNA or RNA, specific proteins (such as NMP22 or Galectin-1), and relevant epigenetic alterations, with high sensitivity and specificity. In many cases, they outperform conventional clinical tests, offering short analysis times (less than one hour), detection in the femtomolar to picomolar range, and direct applicability to urine samples without the need for complex processing. Although many label-free detection techniques demonstrate high analytical sensitivity and specificity in preclinical or pilot studies, their clinical validation across large and diverse patient populations remains limited.

This review aims to provide an exploratory perspective, summarizing primarily preclinical and early translational evidence on label-free detection technologies for NMIBC, and highlighting their potential for future clinical applications.

## 2. Raman Spectroscopy (RS)

The fundamental principle of Raman spectroscopy (RS) lies in the interaction between incident light (typically a laser) and the molecules of a sample, producing a phenomenon known as Raman scattering, which reflects specific vibrational modes of the molecules [9].

Surface-enhanced Raman scattering (SERS) is an advancement of conventional Raman spectroscopy. This technique amplifies the Raman signal by a factor of 10^5^ to 10^6^ when scattering occurs on the surface of certain metallic nanoparticles or nanostructured noble metal surfaces [10]. As a result, SERS enables highly precise detection of the unique “fingerprints” of chemical structures. Due to its high sensitivity and specificity, both RS and SERS have been widely applied in medicine and other scientific disciplines. Urine, an easily accessible biological fluid, is particularly relevant for disease diagnosis, especially in conditions affecting the urinary system [11].

**Figure 1:**
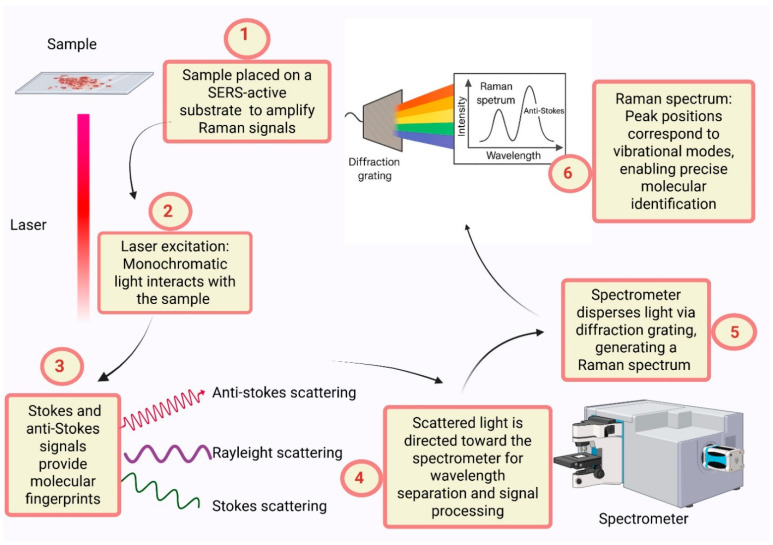
Schematic representation of Surface-Enhanced Raman Spectroscopy (SERS) applied to bladder cancer detection. A laser interacts with a biological sample placed on a SERS-active substrate, generating Rayleigh, Stokes, and anti-Stokes scattering. The scattered light is collected and directed through a diffraction grating to a spectrometer, where the Raman spectrum is recorded and analyzed. Figures were created with BioRender, web-based version accessed on 30 July 2025.

Owing to its molecular precision and high specificity, RS offers a powerful alternative for detecting chemical compounds, specific molecules, antigens, and cellular components [10]. Various diagnostic strategies based on Raman spectroscopy have been developed, including the detection of cancer cell lines, exfoliated cells in urine or their supernatants, and SERS-based methods for identifying antigens or nucleic acids in urinary samples. When combined with computational algorithms such as principal component analysis (PCA), linear discriminant analysis (LDA), random forest (RF), and convolutional neural networks (CNN), the diagnostic accuracy of RS-based approaches is significantly enhanced [11]. A scheme of SERS is shown in Figure 1.

### 2.1. Modulated Raman Spectroscopy (MRS)

Modulated Raman spectroscopy (MRS) addresses the issue of background fluorescence that interferes with Raman signals in biological samples by modulating the laser wavelength [12]. In urine-based analyses, Canetta and colleagues used MRS to distinguish the normal human urothelial cell line (SV-HUC-1) and the bladder cancer cell line (MGH-U1). While conventional Raman spectroscopy achieved 85% classification accuracy, MRS increased the identification accuracy to 97%.

An additional advantage of MRS is its minimal sample-handling requirement: urine spectra can be acquired in just 30–40 s, matching the speed of conventional Raman spectroscopy. These preliminary findings suggest that this label-free approach may hold promise for bladder cancer detection; however, further validation in larger cohorts is needed to confirm its clinical utility.

### 2.2. Wavelength Modulated Raman Spectroscopy (WMRS)

Another optimized version of Raman spectroscopy was developed by Praveen et al., who employed wavelength-modulated Raman spectroscopy (WMRS) to differentiate between the SV-HUC-1 and MGH-U1 cell lines using PCA [13]. This technique modulates the laser wavelength to eliminate interference from background fluorescence.

The method enabled even faster detection—within just 6 s—while achieving acceptable discrimination between the cell lines, as assessed by the signal-to-noise ratio, which reflects the clarity of the signal relative to background noise. WMRS thus proved to be a faster alternative for cell line detection, maintaining sufficient discriminatory power.

### 2.3. Laser Tweezers Raman Spectroscopy (LTRS)

A key limitation of the previous studies is that they compared only two cell lines. To extend Raman-based classification, Tang et al. (2023) integrated optical tweezers with Raman spectroscopy—an approach termed laser-tweezers Raman spectroscopy (LTRS)—to analyze a broader panel of bladder-cancer cell lines (seven in total) [14]. They applied statistical algorithms, including random forest (RF) and PCA, to analyze the Raman spectral data from six bladder cancer cell lines.

The results demonstrated that PCA enabled correct classification rates ranging from 54.4% to 100%, with the invasive T24 cell line identified with 100% accuracy and the cisplatin-resistant T24-CDDPR line at 98.1%. Using the RF algorithm, the classification accuracy ranged from 85.3% to 100%, with similar high performance for these two aggressive cell lines. Notably, less invasive cell lines were more challenging to classify accurately.

LTRS showed encouraging performance in distinguishing invasive and chemotherapy-resistant BC cell lines in a controlled setting, suggesting its potential for the early detection of aggressive tumor phenotypes; however, further validation is needed in clinical samples.

### 2.4. Raman Molecular Imaging (RMI)

Raman microspectroscopic imaging (RMI) has been utilized to collect and analyze Raman spectra from urine supernatants [15]. By monitoring changes in the signal at 1584 cm^−1^, researchers were able to distinguish tumor cells from normal cells. The trained classification model achieved a sensitivity of 92% and a specificity of 90.5% in the validation set. Moreover, RMI demonstrated an accuracy of 73.9% in identifying low-grade tumors and even higher accuracy for high-grade tumors.

### 2.5. Chemometric Urinalysis Based on Raman Spectroscopy (Rametrix™)

Huttanus et al. developed a urinalysis system known as Rametrix™, which achieved a diagnostic accuracy of 80.4% for bladder cancer [16]. The system demonstrated a sensitivity of 82.4% and a specificity of 79.5% in distinguishing high-grade from low-grade tumors.

### 2.6. Raman Spectroscopy Combining Coherent Anti-Stokes Raman Scattering (CARS) and Second Harmonic Generation (SHG)

Using an integrated CARS–SHG platform, Yosef et al. trained a random-forest (RF) model to discriminate tumor cells from normal urothelial cells. Cancerous cells exhibited a loss of Raman signals associated with glycogen—specifically at 941 cm^−1^, 861 cm^−1^, and 482 cm^−1^, and an increase in signals related to lipids and nucleic acids. Based on these spectral differences, the model achieved 100% accuracy in identifying high-grade urothelial carcinoma [17].

### 2.7. SERS Model in Rat Urine

A non-invasive and rapid diagnostic model for early BC detection was developed by combining SERS spectra of urine samples from rats with in situ BC and healthy controls, along with endoscopic findings [18]. BC diagnosis was confirmed using microendoscopy with 5-aminolevulinic acid (5-ALA), followed by histopathological validation. Urine samples were analyzed using a SERS chip composed of zinc oxide coated with gold, featuring a nanoporous structure. For each sample, twenty Raman nano-spectra were acquired within the 450–2250 cm^−1^ range.

PCA and partial least squares discriminant analysis (PLS-DA) were applied, with the combined PCA–PLS-DA approach achieving diagnostic accuracy of ≥99.6% and an area under the curve (AUC) >0.996. These preclinical findings suggest that this label-free SERS approach may serve as a promising tool for sensitive detection of bladder cancer in early stages, although further validation in human samples is required. Translational studies in humans reinforce these pre-clinical results. Lu et al. introduced the concept of “SERSomes”—spectral sets acquired from a single urine droplet—to screen for low-grade bladder cancer, attaining 89% diagnostic accuracy and 90% grade-stratification accuracy in under five minutes [19]. Concurrently, Zhong et al. used label-free urine SERS and PLS-DA to discriminate non-muscle-invasive from muscle-invasive disease with AUC 0.95 and overall accuracies of 97.7% (healthy vs. cancer) and 96.3% (NMIBC vs. MIBC) [20].

Collectively, these reports confirm that urine-based SERS, especially when paired with machine-learning classifiers, delivers rapid, cost-effective and highly sensitive detection across the full biological spectrum, supporting its progression toward point-of-care clinical use.

### 2.8. Application of SERS with Statistical Algorithms

Xi Bai et al. employed SERS to evaluate the performance of three statistical algorithms: PCA–LDA, PLS–RF, and PLS–SVM [21]. Plasma samples from 26 individuals—including BC patients, renal cancer patients, and cancer-free controls—were mixed with silver nanoparticles to generate SERS spectra in the 400–1800 cm^−1^ range. Significant differences in Raman peak intensities were observed between the groups, reflecting the underlying biochemical variations associated with cancer. These spectral differences enabled discrimination of BC and renal cancer patients from healthy individuals. Although this study included only 26 individuals distributed across different analytical groups, it demonstrated high accuracy in distinguishing plasma samples from early-stage and advanced bladder cancer patients versus cancer-free controls. However, larger studies are necessary to validate these findings and support their clinical translation.

## 3. Label-Free Blue Light Cystoscopy

The standard of care for BC diagnosis is white light cystoscopy (WLC) [2]. However, this technique has limited sensitivity for detecting small lesions, benign tissue, and flat lesions—such as those characteristic of carcinoma in situ (CIS). It has been reported that up to 45% of residual tumors may remain undetected using WLC alone [2].

Several enhanced detection techniques have been investigated to improve diagnostic performance. One of the most established is blue light cystoscopy (BLC), which utilizes an exogenous contrast agent—hexaminolevulinate (Cysview^®^)—that selectively accumulates in malignant tissue and fluoresces as bright red patches against a blue background. This approach has been shown to increase the detection rate of high-grade tumors by up to 43% compared to WLC [2].

However, BLC is associated with high implementation costs because of the need for specialized equipment, contrast agents, and dedicated procedural time. Moreover, fewer than 5% of hospitals in the United States currently have access to BLC technology [22].

In this context, Chuang et al. developed a digital staining method to transform WLC images into BLC-like images using artificial intelligence (AI) [23]. They recruited 31 patients and recorded paired WLC and BLC videos. Using an image-to-image (I2I) translation model based on generative adversarial networks (GANs), they applied the Density Changing Regularized Unpaired Image Translation (DECENT) method to digitally “stain” WLC images. This AI-generated transformation achieved an accuracy of 80.58% when compared to true BLC images.

Although further validation is needed before clinical implementation, this AI-based approach offers a promising, cost-effective alternative that could significantly enhance diagnostic capabilities in settings where BLC is not available.

## 4. Impedance Sensor for HTB-9

Hosseini et al. (2017) developed a label-free microfluidic device for the detection and quantification of bladder tumor cells (5637 HTB-9 cell line) by measuring electrical impedance, eliminating the need for molecular markers or biorecognition elements [24]. The system differentiated tumor cells from leukocytes based on membrane capacitance (MC): cancer cells exhibited impedance changes ≥80%, whereas T and B lymphocytes showed changes between 20 and 35%, and empty channels registered <5%.

MC values are significantly higher in bladder cancer cells—up to 50 mF/m^2^—compared to T lymphocytes (16 mF/m^2^) and B lymphocytes (14 mF/m^2^), with statistically significant differences (*p* < 0.001). The device can detect one tumor cell among eleven leukocytes in a 1 mL urine sample, achieving a capture efficiency of 80–90% depending on cell concentration. The optimal operating flow rate is 80 µL/min, and the entire analysis is completed in less than one hour, highlighting the system as a rapid, cost-effective, and non-invasive diagnostic tool for bladder cancer detection in urine samples.

## 5. MALDI-TOF-MS (Matrix-Assisted Laser Desorption/Ionization—Time of Flight Mass Spectrometry)

Two studies have demonstrated the utility of label-free MALDI-TOF-MS for the early and non-invasive detection of NMIBC.

Ding et al. [25] used MALDI-TOF-MS combined with weak cation exchange magnetic beads (WCX-MB) to analyze serum samples from 67 bladder cancer patients. Five differentially expressed peptide peaks (*m*/*z* 1954.9, 2081.0, 3938.3, 3946.5, 4268.8) were used to build a *k*-nearest neighbors model. The diagnostic algorithm achieved 92.31% sensitivity in early-stage bladder cancer and 90.0% sensitivity in low-grade tumors, indicating strong potential for label-free serum-based NMIBC detection.

Sousa et al. [26] extended the MALDI-TOF-MS approach to non-invasive urine and saliva samples, targeting biomarker discovery for cancer diagnosis. Using PCA and hierarchical clustering of mass spectral fingerprints, they identified distinct peptide/protein signatures between bladder cancer patients and healthy controls. Their findings support the feasibility of MALDI-TOF-MS as a rapid, label-free platform for detecting NMIBC-specific molecular patterns in biofluids, highlighting its potential for minimally invasive cancer screening.

Together, these studies reinforce the role of MALDI-TOF-MS in the label-free detection of NMIBC, offering complementary approaches using both serum and urinary matrices.

## 6. Biosensors

Biosensors are analytical devices that integrate a biological recognition element—such as antibodies, aptamers, or enzymes—with a physicochemical transducer that converts the interaction with a target analyte into a quantifiable signal, which may be electrical, optical, thermal, or mechanical [8]. Traditional methods for biomolecule detection often rely on labeled molecules—such as fluorophores, enzymes, or radioactive isotopes—that bind to the analyte to enable detection. However, these procedures can be complex and labor-intensive, potentially alter binding sites, and, in some cases, reduce specificity or affinity. They also involve additional costs and longer processing times.

Label-free biosensing techniques offer several advantages over traditional urine cytology in detecting NMIBC. While cytology is highly specific, it has poor sensitivity for low-grade tumors, often missing early-stage or flat lesions such as carcinoma in situ (sensitivity as low as 20–50%) [5]. Label-free biosensors enable real-time, quantitative, and highly sensitive detection of molecular or cellular changes without the need for dyes, antibodies, or enzymatic labels, reducing operator dependence and improving early detection accuracy [8].

This approach enables the direct detection of analytes based on their intrinsic properties—such as molecular mass, electrical charge, impedance, refractive index, or dielectric characteristics—without requiring chemical modification of the target molecule.

### 6.1. Optical Biosensors

#### 6.1.1. Quantitative Interferometric Label-Free Imaging Flow Cytometry

This is a cellular analysis technique that combines flow cytometry with quantitative phase interference microscopy. Unlike traditional flow cytometry, this method does not require fluorescent markers or labeling to identify and classify cells. Instead, it relies on intrinsic variations in the refractive index (RI), which reflect the internal structure of cells. Phase interference microscopy measures the optical path delay (OPD) of light as it passes through cells, a parameter directly related to their refractive index.

Cells are passed through a microfluidic system and analyzed using interferometric phase microscopy, producing quantitative images from which both optical and morphological features are extracted. These features are then used to classify cells into distinct populations—such as healthy versus malignant—using machine learning algorithms. RI and OPD have previously been validated as biomarkers in various cell types, including prostate cells [27].

One study demonstrated the utility of combining label-free Quantitative Interferometric Flow Cytometry (QIFC) with urine samples. This non-invasive method was evaluated for its ability to detect malignant cells in urine through label-free analysis. The study included eight bladder carcinoma cell lines, normal urothelial cells, and urine samples from patients with bladder cancer and healthy volunteers. Cells were processed through microfluidic channels and imaged using phase interference microscopy to generate OPD maps. Twenty morphological and optical features were extracted per cell. Using two machine learning algorithms—convolutional neural networks (CNN) and XGBoost—the system achieved binary classification with sensitivity greater than 98%, underscoring its potential as a diagnostic and surveillance tool for bladder cancer [28].

Surface Plasmon Resonance (SPR) is a real-time, label-free optical sensing technique that detects biomolecular interactions by measuring refractive index changes at a metal-dielectric interface. In their comprehensive review, Gade et al. [29] highlight SPR’s exceptional suitability for early cancer detection, emphasizing its capacity to identify low-abundance biomarkers—including ctDNA, exosomes, and proteins—from bodily fluids such as urine. While not restricted to bladder cancer, their analysis underscores SPR’s high analytical sensitivity and non-invasive applicability, features that closely align with the clinical demands of NMIBC screening.

In a more focused perspective, Das et al. [30] explore recent advances in SPR biosensor design, including improvements in surface functionalization and nanomaterial-based signal amplification. They report diagnostic performances reaching up to 94% sensitivity and 90% specificity for urinary cancer biomarkers, positioning SPR as one of the most reliable and reproducible label-free approaches currently available. Compared to other techniques such as SERS or interferometric flow cytometry—which, despite offering higher theoretical sensitivity, still face challenges in standardization and clinical scalability—SPR stands out for its robustness, ease of integration, and validated clinical potential in early NMIBC detection.

#### 6.1.2. Label-Free DNA Sensor for Detection of Bladder Cancer Biomarkers in Urine

Similarly, a label-free optical biosensor was developed based on silicon microring resonators for the direct detection of urinary DNA mutations associated with low-grade bladder cancer [31]. The sensor employs surface-immobilized DNA probes that specifically recognize mutations such as FGFR3 S249C and HRAS G13R. Specific hybridization between the probe and target DNA induces a change in the local refractive index, which in turn shifts the optical resonance peak of the microring—measured without the need for labels or dyes.

In tests using DNA derived from actual urine samples, the biosensor achieved sensitivity of 83% and a specificity of 89% for detecting mutated FGFR3. The device demonstrated strong stability and diagnostic accuracy even in unpurified urine, highlighting its potential as a non-invasive tool for screening and monitoring low-grade bladder cancer.

### 6.2. Electrochemical Biosensors

Electrochemical sensors developed for NMIBC detection commonly target NMP22, survivin, and cytokeratins, as well as select microRNAs. These urinary biomarkers are clinically relevant due to their association with urothelial cell turnover, tumor proliferation, and recurrence risk [32]. Their presence in urine provides a non-invasive means to monitor disease activity. Electrochemical platforms offer enhanced sensitivity for these targets, particularly in early-stage and low-grade NMIBC, where conventional tests like cytology and ELISA have limited performance.

#### 6.2.1. NMP22 Detection

NMP22 is a key biomarker for bladder cancer detection, traditionally measured using ELISA or rapid tests such as BladderChek^®^, which require labeled reagents and exhibit limited sensitivity for detecting trace biomarker levels in early-stage disease [32]. In contrast, a label-free electrochemical immunosensor was developed using a 2D/3D hybrid nanocomposite platform (rGO-TEPA@Cu-MOFs@SiO_2_@AgNPs) to enable ultra-sensitive, direct detection of NMP22 in urine without the need for labeling.

The sensor was constructed using an amplification strategy based on hybrid nanomaterials, combining functionalized reduced graphene oxide (rGO-TEPA) with copper-based metal–organic frameworks (Cu-MOFs) coated with silica and silver nanoparticles. This configuration significantly improved anti-NMP22 antibody immobilization and enhanced electrode conductivity. The resulting system achieved a detection limit as low as 33.33 fg/mL and demonstrated high selectivity in human urine samples.

The synergistic combination of materials provides an increased electroactive surface area and greater system stability. When validated against ELISA, the sensor showed a strong correlation (r = 0.998), supporting its potential clinical applicability as a non-invasive and highly sensitive diagnostic tool.

While this configuration enhances the analytical performance of the system, its ability to detect NMP22 at low concentrations in urine samples suggests potential for improving early-stage NMIBC diagnosis with greater sensitivity than conventional methods such as ELISA or cytology. This could facilitate more accurate, non-invasive monitoring in outpatient settings.

#### 6.2.2. Label-Free Impedimetric Immunosensor for Galectin-1

Galectin-1 (Gal-1) is a lectin that is overexpressed in high-grade BC [24]. Chuang et al. (2016) developed a label-free immunosensor consisting of gold interdigitated microelectrodes on a chip, where alumina nanoparticles functionalized with anti-Gal-1 antibodies were immobilized using positive dielectrophoresis (p-DEP) [23].

The immunosensor exhibited a concentration-dependent response to T24 cell lysate (grade III), with a detection limit of 0.0078 mg/mL in phosphate-buffered saline (PBS), as well as in artificial and human urine. At this concentration, the normalized impedance variation was approximately 4%, increasing to 56% at 0.25 mg/mL—a 76-fold signal increase compared to the control without lysate (0.69%).

Moreover, the sensor was able to distinguish between tumor grades: at the same concentration (0.03125 mg/mL), T24 cell lysate produced a 17.1% impedance variation, compared to only 1.3% with RT4 cell lysate (grade I), with a statistically significant difference (*p* < 0.001). These findings were validated by Western blot analysis, confirming differential expression of Gal-1 between high- and low-grade tumors.

#### 6.2.3. Electrochemical Impedance Spectroscopy (EIS) Applied to Genosensors

Pursey et al. (2017) developed a label-free electrochemical genetic biosensor employing porphyrin-modified DNA hairpin probes to target three commonly methylated bladder cancer genes: CDH1, DAPK, and RARβ [33]. Specific hybridization between tumor-derived DNA and the probes induced measurable changes in impedance, which were detected using electrochemical impedance spectroscopy (EIS).

The biosensor achieved a detection limit of 250 fM, which falls within the clinically relevant concentration range of cell-free DNA in urine. Specificity was validated by introducing four-base mutations into the target sequence, resulting in a fivefold reduction in the electrochemical signal compared to the fully complementary probe (*p* < 0.01).

The sensor enabled simultaneous detection of all three genetic biomarkers in mixed solutions and demonstrated comparable performance in both citrate buffer and synthetic urine, confirming its robustness. The total analysis time was approximately 20 min for 5 µL samples, positioning this platform as a rapid, sensitive, and label-free tool for non-invasive bladder cancer screening.

### 6.3. Physical Biosensors

#### Label-Free Long Non-Coding RNA Assay on String for Bladder Cancer Detection

Recently, a label-free biosensor based on nanopore technology was developed for the detection of long non-coding RNAs (lncRNAs), using UCA1 as a model biomarker for bladder cancer. The system employed a single-stranded DNA detector with complementary capture arms capable of recognizing long RNA sequences (>2 kb). When UCA1 molecules bound to the detector passed through the nanopore, they generated larger and longer ionic current blockades compared to the unbound detector [34].

In addition, characteristic multi-step current drop events were observed, reflecting complex secondary structures of the UCA1 transcript during translocation. This system successfully detected individual UCA1 molecules under controlled experimental conditions, highlighting its potential as a sensitive, specific, and label-free diagnostic tool for early bladder cancer detection using urine samples.

## 7. Discussion

Label-free methods represent a promising alternative to conventional diagnostic tools used in bladder cancer. Its ability to identify relevant biomarkers—such as exfoliated cells, specific proteins, methylated DNA, or non-coding RNA—without the need for labeled reagents allows for simplified sample processing, reduced costs, and improved clinical applicability, especially in settings with limited access to advanced technology.

What did we find? While both SERS and SPR represent powerful label-free detection strategies, their degree of clinical investigation in bladder cancer differs. SERS has been more extensively studied specifically in the context of bladder cancer, particularly for NMIBC. Several studies have applied SERS to urine samples for the detection of tumor cells, metabolites, and nucleic acids, demonstrating excellent sensitivity and classification accuracy. However, the technique still faces significant challenges in clinical translation due to variability in substrate fabrication and limited standardization across laboratories. In contrast, SPR is a more mature and clinically validated biosensing platform, widely used for the detection of low-abundance biomarkers in various cancers. Although fewer studies have focused on the specific application of SPR to NMIBC, the technique offers superior reproducibility, real-time detection capabilities, and diagnostic performance up to 94% sensitivity and 90% specificity. Thus, while SERS currently holds a broader research base in bladder cancer, SPR may offer greater translational potential for future clinical implementation.

While label-free detection technologies demonstrate high sensitivity and specificity in experimental settings, their clinical translation faces several barriers. Challenges include the need for technical validation under clinically relevant conditions, including testing with sufficiently large and diverse patient cohorts. Without such validation, results from small-scale laboratory studies may not reliably translate into practical, point-of-care diagnostic tools. For example, methods based on advanced optical detection such as SERS and WMRS require specialized equipment and technical expertise that may not be readily available in all clinical environments. Moreover, the lack of large-scale clinical validation studies limits their immediate applicability.

In terms of clinical translation, compact electrochemical biosensors offer distinct advantages over optical methods such as SERS. Their low manufacturing cost, portability, and minimal instrumentation make them highly compatible with point-of-care applications and scalable deployment. By contrast, SERS platforms require complex optical components, precise alignment, and specialized nanostructures, which limit their standardization and integration into routine clinical settings. Although SERS may offer higher analytical sensitivity, electrochemical platforms remain more feasible for widespread adoption, especially in resource-constrained environments. Table 1 shows the label-free detection technologies for NMIBC with reported sensitivity and specificity.

The gap between preclinical promise and clinical utility can be attributed to the nascent stage of these technologies and the technical complexities involved. Greater investment in translational research, validation protocols, and practical integration strategies will be essential to bridge this gap.

It is important that the adoption of label-free biosensors in point-of-care settings still faces specific technological and clinical barriers. These include challenges in sensor standardization, sensitivity to sample matrix variability (particularly in urine), and the need for clinically validated, scalable manufacturing processes. Furthermore, integration into routine diagnostics requires cost-effective miniaturization and regulatory approval—issues that remain unresolved in most current platforms. These factors must be addressed to ensure robust, reproducible, and clinically deployable systems for NMIBC detection.

Beyond sensitivity and specificity, key analytical parameters—such as limit of detection, analysis time, and extent of clinical validation—critically influence the real-world adoption of label-free biosensing platforms. A sufficiently low limit of detection is essential to detect trace levels of biomarkers typical in early-stage NMIBC, while rapid turnaround times enhance applicability in outpatient or point-of-care settings. However, even with optimal analytical performance, lack of multicenter clinical validation remains a major obstacle for regulatory approval and routine implementation. These combined metrics ultimately determine the clinical readiness and translational value of each platform.

Another crucial aspect that directly influences reproducibility and accuracy is the implementation of standardized sample handling systems. These devices minimize pre-analytical variability by ensuring consistency in parameters such as sample volume, filtration, and storage conditions—factors particularly relevant in complex biological fluids like urine. For label-free diagnostic platforms, where signal responses are subtle and highly sensitive to matrix effects, standardized handling—often enabled by integrated microfluidic or cartridge-based formats—is essential to achieve reliable, reproducible, and clinically transferable results.

In conclusion, the reviewed technological advances highlight the potential of label-free technologies as non-invasive, accurate, and scalable tools for the diagnosis and follow-up of NMIBC. Their future implementation could shift the diagnostic paradigm toward more accessible, rapid, and patient-centered methods, facilitating outpatient monitoring and early detection. However, further studies are needed to evaluate the feasibility of these techniques in clinical practice.

## 8. Future Directions

Label-free detection strategies for NMIBC show increasing promise, but several challenges remain before their clinical translation. Key priorities include the standardization of protocols, particularly in biosensor fabrication and sample processing, and the validation of performance across large, diverse patient cohorts.

Technological improvements—such as microfluidics, machine learning-assisted analysis, and hybrid platforms—could enhance sensitivity, reproducibility, and integration into clinical workflows. The use of non-invasive samples like urine and saliva, combined with multiplexed detection of biomarkers or biophysical parameters, is expected to support more personalized and less invasive monitoring of NMIBC.

Continued interdisciplinary collaboration and robust clinical trials will be essential to establish these technologies as practical tools for early diagnosis and surveillance.

## Figures and Tables

**Table 1:** Comparison of label-free detection technologies for NMIBC, ranked by reported diagnostic performance (sensitivity and specificity).

Method	Sensitivity	Specificity	Type/Principle	Analysis Time	Clinical Context	Reference
SERS (Surface-Enhanced Raman Spectroscopy)	99.6%	>99%	Optical (enhanced vibrational spectroscopy)	Seconds to minutes	Diagnostic (urine samples, AI-enhanced)	Zhon et al. 2024 [21]
SERS in rat urine model	≥99.6%	>99%	Optical (SERS + AI)	Minutes	Diagnostic (rat model)	Lee et al. 2024 [18]
SERS + PLS-SVM (plasma)	100%	Not reported	Optical (SERS with statistical analysis)	Minutes	Diagnostic (plasma samples)	Bai et al. 2022 [21]
CARS + SHG (Raman Coherent + Second Harmonic)	100%	Not reported	Optical (non-linear optical methods)	Not specified	Experimental (animal model)	Yosef et al. 2017 [17]
LTRS (Laser Tweezers Raman Spectroscopy)	54–100%	85.3–100%	Optical (optical trapping with Raman)	Minutes	Diagnostic (urine samples)	Tang et al. 2023 [14]
Quantitative Interferometric Flow Cytometry	>98%	Not reported	Optical (label-free interferometric)	Minutes	Diagnostic (proof-of-concept)	Chuang et al. 2020 [35]
MRS (Modulated Raman Spectroscopy)	97%	Not reported	Optical (reduced background Raman)	~30–40 s	Analytical only	Canetta et al. 2011 [12]
Surface Plasmon Resonance (SPR)	Up to 94%	Up to 90%	Optical (surface plasmon resonance)	Real-time	Diagnostic (bladder cancer biomarkers)	Das et al. 2023 [30]
Raman Spectroscopy (RS)	85–97%	90.5%	Optical (vibrational spectroscopy)	Seconds to minutes	Diagnostic (human urine)	Balhara et al. 2023 [11]
RMI (Raman Molecular Imaging)	92%	90.5%	Optical (molecular imaging Raman)	Minutes	Diagnostic (Raman imaging)	Shapiro et al. 2011 [15]
Label-free DNA microring resonator	83%	89%	Optical (resonance shift with hybridization)	~20 min	Analytical (DNA hybridization)	Shin et al. 2013 [31]
Label-free impedimetric Galectin-1 immunosensor	82.1%	80.8%	Electrochemical (impedance-based)	~1 h	Analytical (urine Galectin-1)	Chuang et al. 2016 [23]
Label-free electrochemical DNA sensor (CDH1, DAPK, RARβ)	83–100%	89%	Electrochemical (impedance hybridization)	20 min	Analytical (methylated DNA targets)	Pursey et al. 2017 [33]
Rametrix™ (Chemometric Raman)	80.4%	79.5%	Optical (chemometric Raman analysis)	Minutes	Analytical (urine metabolites)	Huttanus et al. 2020 [16]

## Data Availability

No new data were created or analyzed in this study. Data sharing is not applicable to this article.
